# Reinforcing Calcium Overload via Inflammation‐Mediated Targeting to Amplify Pyroptosis and Antitumor Immunity

**DOI:** 10.1002/advs.202522827

**Published:** 2026-03-23

**Authors:** Yingying Liu, Yang Liu, Qin Fan, Jinqiao Zhang, Kexin Wen, Boyu Yuan, Xianzhou Cai, Ziliang Dong, Lianhui Wang

**Affiliations:** ^1^ Department of Clinical Pharmacy Shandong Key Laboratory of Digital Diagnosis and Treatment of Thoracic Oncology The First Affiliated Hospital of Shandong First Medical University & Shandong Provincial Qianfoshan Hospital Jinan Shandong P. R. China; ^2^ Science and Technology Innovation Center Shandong First Medical University& Shandong Academy of Medical Sciences Jinan Shandong P. R. China; ^3^ Biomedical Sciences College, Shandong Medicinal Biotechnology Centre Shandong First Medical University & Shandong Academy of Medical Sciences Jinan Shandong P. R. China; ^4^ School of Chemistry, Chemical Engineering and Biotechnology Nanyang Technological University Singapore Singapore; ^5^ State Key Laboratory of Flexible Electronics (LoFE) & Institute of Advanced Materials (IAM) Nanjing University of Posts & Telecommunications Nanjing Jiangsu P. R. China

**Keywords:** calcium stress, immune activation, inflammation amplifying, pyroptosis, tumor treatment

## Abstract

Tumor‐associated inflammation presents a promising therapeutic target, yet it remains insufficiently exploited in current nanomedicine approaches. While inflammation‐driven strategies, such as inducing pyroptosis, have the potential to enhance tumor immunity, their limited induction efficiency and poor tumor targeting still pose significant challenges. Herein, we engineer a neutrophil membrane‐camouflaged nanoplatform (RC@NMVs) that exploits tumor inflammation to drive reinforcing calcium dyshomeostasis and pyroptotic amplification. This biomimetic system comprises calcium phosphate (CaP) nanoparticles co‐loaded with the calcium channel blocker, ruthenium red. Upon internalization by tumor cells, the CaP core dissolves within lysosomes, releasing Ca^2^^+^ ions, while ruthenium red inhibits the Ca^2+^ transporting channels, synergistically elevating intracellular Ca^2^^+^ levels. The resulting calcium overload triggers gasdermin‐mediated pyroptosis, characterized by the release of damage‐associated molecular patterns (DAMPs) and pro‐inflammatory cytokines. The amplified inflammatory microenvironment facilitates the recruitment and tumor accumulation of subsequently administered RC@NMVs. In vivo studies demonstrate enhanced tumor enrichment of RC@NMVs compared to non‐inflamed controls, leading to robust pyroptosis and significant tumor inhibition. Moreover, pyroptosis‐driven inflammation elicits durable antitumor immune responses, effectively preventing tumor recurrence and metastasis. In general, this work presents a biomimetic strategy that harnesses inflammation‐mediated tumor targeting to amplify pyroptosis and immune activation, offering a promising approach for effective cancer therapy.

## Graphical abstract

A neutrophil‐membrane‐camouflaged nanoplatform (RC@NMVs) exploits tumor inflammation to target and accumulate in tumors. Intracellular Ca^2+^ overload is triggered via lysosomal CaP dissolution and Ru red‐mediated Ca^2+^ channel blockade, synergistically inducing gasdermin‐mediated pyroptosis. The resulting inflammatory amplification recruits more nanoplatforms, establishes a self‐reinforcing therapeutic cycle, and activates durable antitumor immunity, effectively inhibiting tumor growth, recurrence, and metastasis.

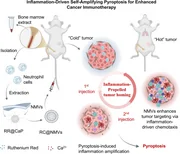

## 1 Introduction

Tumors are intrinsically inflammatory diseases, with their inflammatory nature not only resulting from malignant transformation but also driving tumor progression and immune evasion [1, 2, 3, 4]. Over the past decade, Strategies that exploit the inflammatory microenvironment by harnessing cytokine gradients (e.g., IL‐8, TNF‐α) or immune cell surface markers (e.g., CXCR2 on neutrophils) have been widely explored to enhance tumor‐selective drug delivery, ultimately improving intratumoral accumulation and therapeutic precision [5, 6, 7]. However, their effectiveness is often limited by the inherently heterogeneous and sometimes weak inflammatory status of tumors, particularly in immunologically “cold” tumors with low immune cell infiltration [8, 9, 10]. Recent advances in biomimetic nanoplatforms, particularly those camouflaged with cell membranes such as neutrophil, leukocyte, or tumor cell membranes, have emerged as promising tools for targeted tumor therapy by leveraging natural homing abilities and immune evasion properties [11, 12]. Some designs that utilize neutrophil membrane‐camouflaged carriers offer a promising strategy to further improve tumor homing by harnessing tumor inflammation‐driven chemotaxis [13, 14]. However, the effectiveness of these biomimetic carriers can be limited in tumors with low inflammatory activity, as their chemotactic targeting relies on inflammation‐driven cues [15, 16]. In such cases, insufficient inflammation level compromises targeting efficiency and therapeutic outcome of these inflammation‐dependent strategies.

To overcome this limitation, dynamically manipulating tumor inflammation levels, rather than merely exploiting static features, to create new binding sites and further boost nanoparticle accumulation at tumor sites, has become an urgent priority. For example, pre‐treating tumors with oncolytic viruses or Stimulator of Interferon Genes (STING) agonists can transiently elevate inflammatory cytokine levels, thereby enhancing nanoparticle accumulation [17]. Pyroptosis, or pyroptotic cell death, is a highly inflammatory form of programmed cell death that has emerged as a compelling strategy for cancer treatment [18, 19, 20]. Pyroptosis leads to rapid plasma membrane rupture and the release of pro‐inflammatory cytokines and DAMPs, which can convert immunologically “cold” tumors into “hot” ones, thereby enhancing antitumor immune responses [21, 22]. One key trigger of pyroptosis is intracellular Ca^2^^+^ overload, which perturbs cell homeostasis and activates gasdermin proteins responsible for membrane pore formation [23]. However, current Ca^2^^+^‐based strategies face significant challenges in sustaining effective intracellular calcium overload, particularly due to cellular mechanisms that rapidly efflux excess calcium [24, 25]. These challenges highlight the need for strategies that can amplify Ca^2^^+^‐induced pyroptosis by maintaining intracellular calcium overload, thereby intensifying inflammatory signaling and enhancing immune activation within the tumor microenvironment.

**SCHEME 1:**
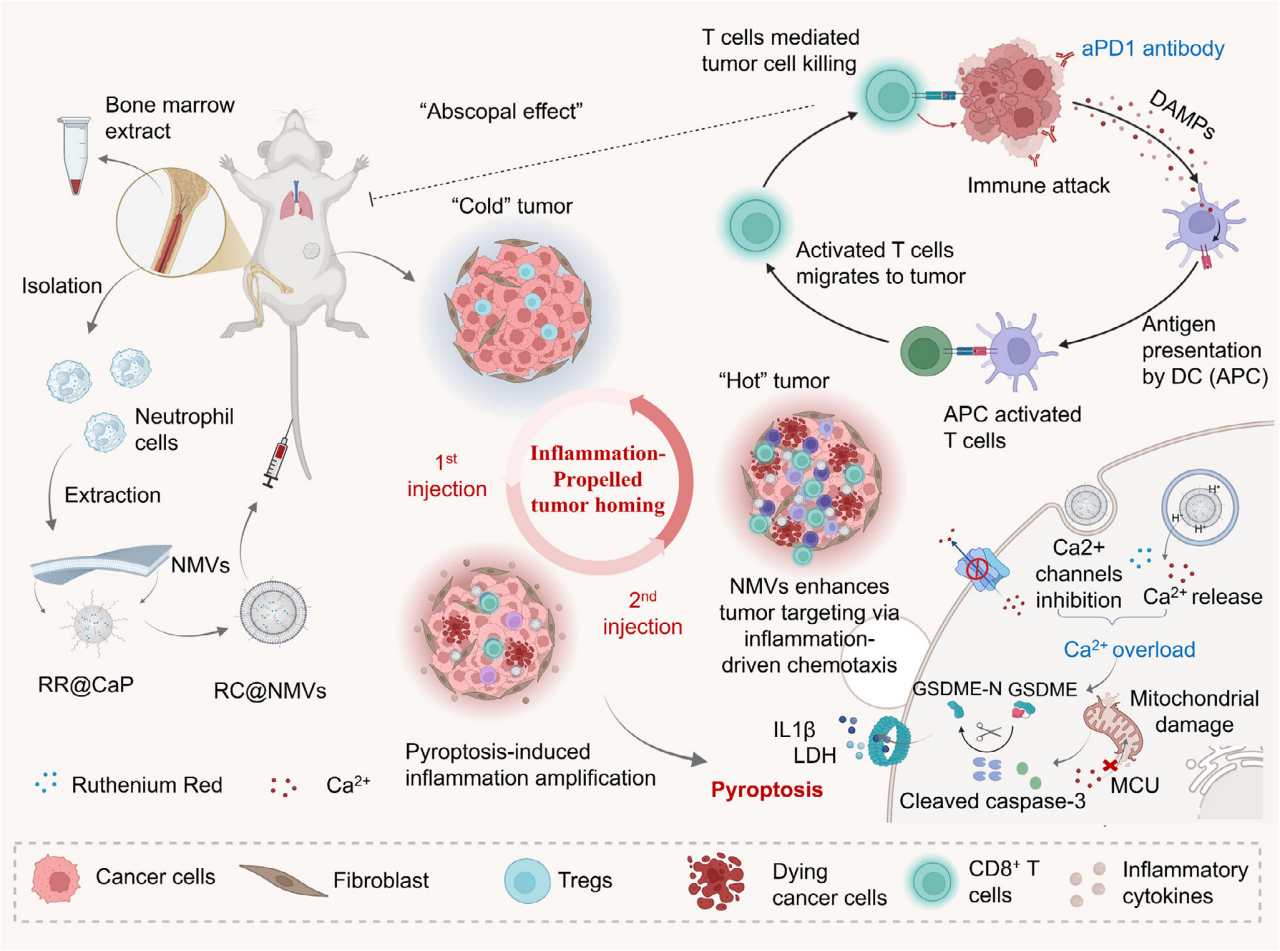
A schematic illustrates the inflammation‐boosted neutrophil mimetics enable self‐enhanced Enable tumor targeting and pyroptotic activation for potent antitumor immunity. The biomimetic RC@NMVs nanoparticles exploit tumor‐associated inflammation for targeted delivery, where their CaP core dissolves in lysosomes to release Ca^2^^+^ while ruthenium red inhibits cellular calcium‐regulating channels, creating intracellular calcium overload. This combined effect triggers gasdermin‐mediated pyroptosis, characterized by cell swelling and release of DAMPs and pro‐inflammatory cytokines that further amplify the tumor inflammatory microenvironment. The enhanced inflammation promotes additional RC@NMVs recruitment, further amplifying pyroptotic cell death. Concurrently, RC@NMVs ‐mediated pyroptosis generates durable antitumor immunity that suppresses both primary tumor growth and metastatic spread. By integrating inflammation‐driven targeting, calcium dyshomeostasis‐induced pyroptosis, and immune activation into a single platform, this strategy overcomes current limitations in tumor‐specific delivery and pyroptosis induction efficiency for more effective cancer treatment.

In this study, we proposed a self‐reinforcing nanotherapeutic strategy to locally amplify tumor inflammation by leveraging calcium stress‐induced pyroptosis, thereby activating antitumor immunity and enhancing therapeutic outcomes. The engineered nanoplatform consisted of CaP nanoparticles, which are highly biocompatible and degrade in acidic lysosomes to release Ca^2+^, thereby initiating intracellular calcium accumulation, loaded with the calcium channel inhibitor ruthenium red, a calcium channel inhibitor, disrupts intracellular calcium homeostasis [26, 27, 28], and cloaked in neutrophil‐derived membrane vesicles (RC@NMVs). Upon cellular uptake, the RC@NMVs dissolved in the acidic lysosomal environment, releasing Ca^2^^+^ ions, alongside ruthenium red, which then inhibited cellular calcium‐regulating channels to disrupt homeostasis and induce calcium overload. This dual mechanism synergistically promoted sustained intracellular Ca^2^^+^ overload, thereby efficiently activating gasdermin‐mediated pyroptosis. The resulting inflammatory cell death led to the release of DAMPs and pro‐inflammatory cytokines, which amplified local tumor inflammation and, leveraging the inflammation‐responsive chemotactic properties of the neutrophil membrane, facilitated the recruitment and tumor accumulation of subsequently administered RC@NMVs nanoparticles. In vitro studies confirmed robust pyroptotic activity and immune activation, while in vivo results demonstrated significantly enhanced tumor accumulation, effective tumor suppression, and durable immune responses that prevented tumor recurrence and metastasis (Scheme 1). This work highlights a biomimetic, inflammation‐amplifying strategy, offering a promising approach for overcoming the limitations of immunologically “cold” tumors.

## 2 Results and Discussion

### 2.1 Preparation and Characterization of RC@NMVs

**FIGURE 1:**
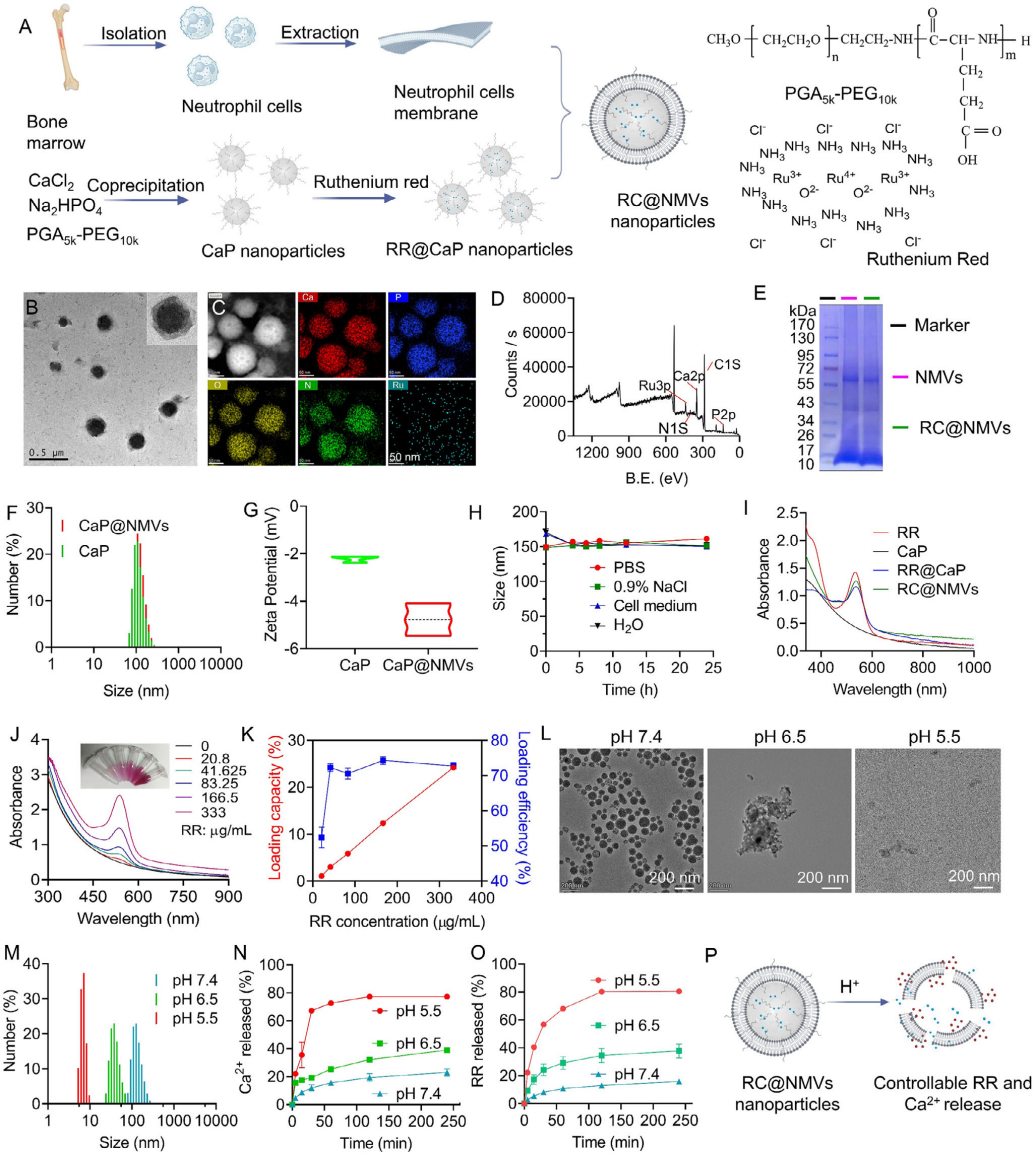
Preparation and characterization of RC@NMVs. (A) Schematic illustration of the fabrication process of RC@NMVs. (B,C) TEM image (B) and corresponding elemental mapping (C) of RC@NMVs. (D) XPS spectra of RC@NMVs, demonstrating the elemental composition. (E) SDS‐PAGE analysis of neutrophil membranes and RNC@NMVs. (F,G) Size distribution (F) and zeta potential (G) of CaP and RC@NMVs, indicating successful membrane coating. (H) Time‐dependent size changes of RC@NMVs in 0.9% NaCl, PBS, and cell culture medium (n = 3), indicating their colloidal stability under different physiological conditions. (I) UV‐vis absorption spectra of RR, CaP, CaP@NMVs, and RC@NMVs. (J) UV‐vis absorption spectra of RC@NMVs with varying amounts of Ruthenium red, showing concentration‐dependent increases in characteristic absorbance. (K) Loading efficiency of Ruthenium red in RC@NMVs at different feeding concentrations. (L) Typical TEM images of RC@NMVs incubated at different pH values (7.4, 6.5, and 5.5) (n = 3), illustrating morphology changes under varying acidic conditions. (M) DLS measurements showing the hydrodynamic size changes of RC@NMVs after incubation at different pH values (7.4, 6.5, and 5.5) (n = 3). (N,O)Time‐dependent release profiles of Ca^2^^+^ ions (N) and Ruthenium red (O) from RC@NMVs under different pH conditions (7.4, 6.5, and 5.5) (n = 3). (P) scheme showing the pH‐responsive RR and Ca^2+^ release from RC@NMVs. Data are expressed as mean values with standard error of the mean (SEM) based on at least triplicate independent measurements.

RC@NMVs were fabricated via a sequential three‐step process. Initially, CaP cores were synthesized using poly(γ‐glutamic acid)‐b‐poly(ethylene glycol) (PGA_5K_‐PEG_10K_) block copolymers as templates, following a mineralization method. The cationic ruthenium red (RR) was loaded into the CaP nanoparticles via electrostatic interaction and physical adsorption. Concurrently, neutrophil membrane‐derived vesicles were prepared according to established protocols [29]. Finally, the RR‐loaded CaP nanoparticles were mixed with the membrane vesicles and passed through a mini‐extruder, facilitating membrane fusion and yielding core‐shell structured neutrophil membrane‐cloaked nanoparticles (RC@NMVs) (Figure 1A). The physicochemical properties of RC@NMVs were then carefully characterized. Transmission electron microscopy (TEM) revealed that, in contrast to the uncoated CaP nanoparticles (Figure S1), RC@NMVs exhibited a distinct core‐shell architecture with a membrane shell thickness of ∼15.6 nm (Figure 1B). Elemental mapping and x‐ray photoelectron spectroscopy (XPS) confirmed the presence of key elements associated with both the RR@CaP core and characteristic membrane components (e.g., C/N/O) (Figure 1C,D). Agarose gel electrophoresis revealed that RC@NMVs and isolated neutrophil membrane vesicles exhibited similar migration patterns, suggesting the existence of membrane‐associated proteins after coating (Figure 1E). Moreover, dynamic light scattering (DLS) measurements indicated a hydrodynamic size increase from 110.2 to 125.3 nm after coating, while zeta potential shifted from −2.2 to −4.8 mV, further confirming the presence of the negatively charged neutrophil membrane on the nanoparticle surface (Figure 1F,G). The colloidal stability of RC@NMVs in various physiological solutions was confirmed by DLS, which showed minimal changes in hydrodynamic diameter over 24 h, indicating good stability (Figure 1H). Moreover, stability tests conducted in higher‐concentration serum environments (20% and 30% FBS) further demonstrated consistent particle size and dispersion over 48 h (Figure S2), supporting the suitability of RC@NMVs for in vivo applications.

Subsequently, UV‐vis absorption spectroscopy showed a characteristic peak at 550 nm corresponding to ruthenium red, confirming its successful loading into the CaP nanoparticles (Figure 1I; Figure S3). The absorbance intensity increased with increasing feeding concentration of ruthenium red, and the loading efficiency reached up to ∼72.3%, indicating efficient encapsulation by the CaP (Figure 1J,K). Given the acid‐responsive dissociated nature of the CaP core, we next investigated the pH‐dependent disassembly behavior of RC@NMVs. Under acidic conditions (pH 5.5), TEM imaging revealed structural disruption of the RC@NMVs, and DLS measurements showed a significant decrease in hydrodynamic diameter, suggesting their disintegration (Figure 1L,M). Concurrently, inductively coupled plasma mass spectrometry (ICP‐MS) analysis revealed a time‐dependent release of Ca^2^^+^ ions, with ∼77.2% released after 4 h of incubation at pH 5.5, compared to only ∼22.3% at pH 7.4 under the same conditions (Figure 1N). Similarly, the release profile of ruthenium red (RR) was monitored via UV‐vis absorption spectroscopy at 550 nm, showing a gradual increase in absorbance over time. After 4 h, the cumulative RR release reached ∼80.5% at pH 5.5, whereas only ∼15.9% was released at pH 7.4 (Figure 1O). These results demonstrate that RC@NMVs enable efficient pH‐triggered Ca^2^^+^ ions and RR release under acidic conditions (Figure 1P).

### 2.2 Intracellular Calcium Overload‐Mediated Cytotoxicity Induced by RC@NMVs

**FIGURE 2:**
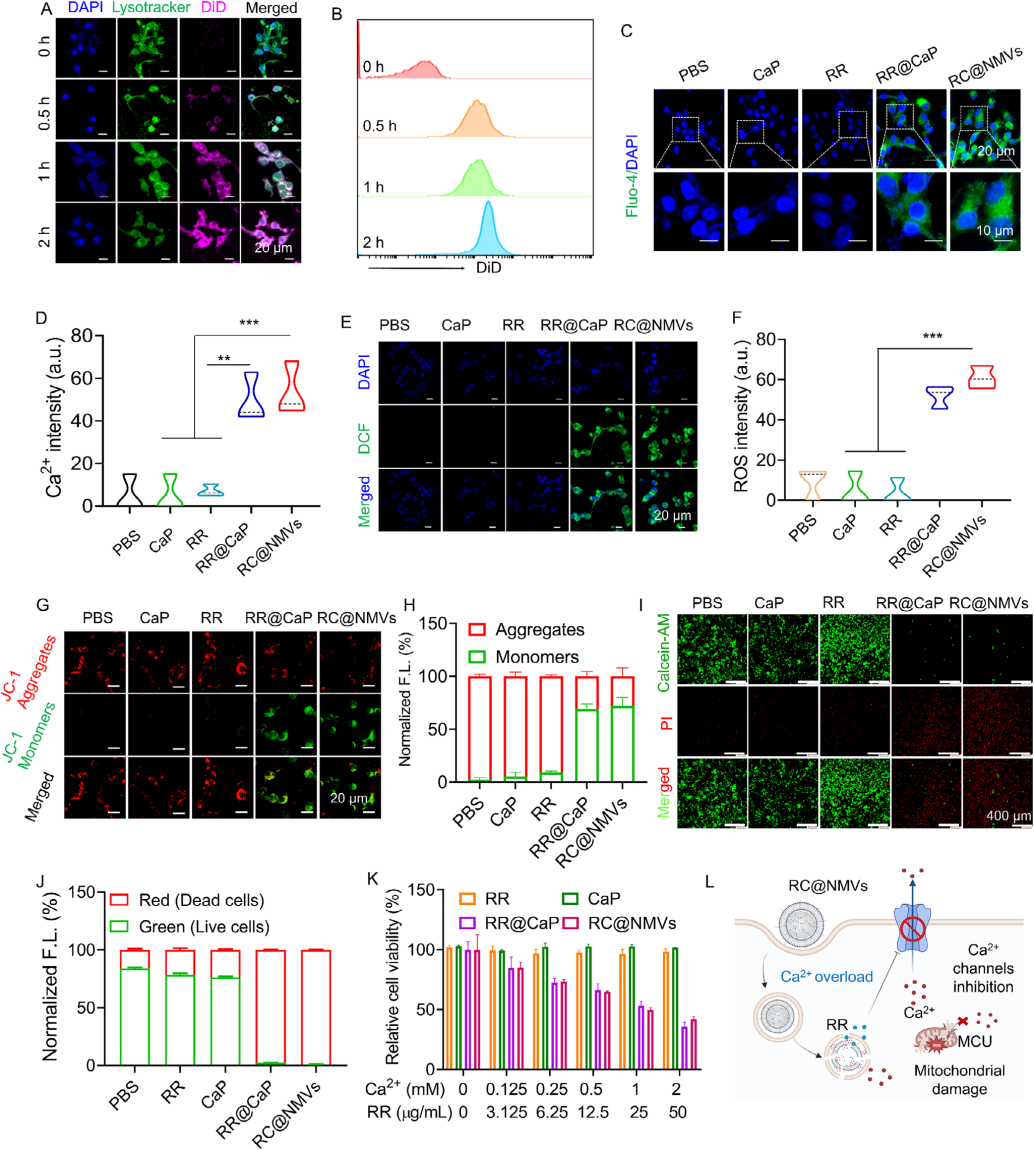
RC@NMVs induce cytotoxicity through intracellular calcium overload. (A,B) Time‐dependent cellular uptake of RC@NMVs by confocal fluorescence imaging (A) and flow cytometry (B). (C,D) Confocal fluorescence images (C) and corresponding quantitative analysis (D) of intracellular Ca^2^^+^ levels stained with Fluo‐4 after different treatments. (E,F) Confocal fluorescence images (E) and corresponding quantitative analysis (F) of intracellular ROS levels stained with DCFH‐DA after different treatments. (G,H) Confocal fluorescence images (G) and corresponding quantitative analysis (H) of mitochondrial membrane potential using JC‐1 staining after different treatments. (I,J) Live/dead cell staining with Calcein‐AM and PI by confocal microscopy (I) and corresponding quantitative analysis (J) after different treatments. (K) Relative cell viability of CT26 cells after different treatments by MTT assay (n = 6). (L) Schematic illustration of RC@NMVs inducing cytotoxicity via intracellular calcium overload. Data are expressed as mean ± SEM. Statistical significance was analyzed using one‐way ANOVA with Tukey's multiple comparison test; ^*^ *p* < 0.05, ^**^ *p* < 0.01, ^***^ *p* <0.001.

We subsequently investigated the potential of RC@NMVs to synergistically modulate intracellular calcium levels through the co‐release of Ca^2^^+^ and RR, thereby inducing calcium overload and triggering tumor cell death. Confocal laser scanning microscopy (CLSM) and flow cytometry were employed to assess the cellular uptake of RC@NMVs over time, confirming efficient and time‐dependent internalization by CT26 cells (Figure 2A,B). Then, intracellular calcium levels were analyzed using the calcium‐sensitive fluorescent probe Fluo‐4 AM. Notably, CT26 cells treated with RC@NMVs or RC (CaP‐RR) exhibited significantly enhanced green fluorescence compared to those treated with CaP (calcium delivery only) or RR (calcium channel blockade) alone, indicating that the synergistic effect for amplifying intracellular calcium accumulation (Figure 2C,D). Concurrently, intracellular reactive oxygen species (ROS) generation was monitored using the DCFH‐DA probe. Strong green fluorescence was observed in the RC@NMVs group, suggesting calcium‐induced oxidative stress (Figure 2E,F). Afterward, mitochondrial membrane potential disruption was further evaluated by JC‐1 staining, which revealed a clear red‐to‐green fluorescence shift following RC@NMVs treatment, indicative of mitochondrial depolarization (Figure 2G,H). Based on the above observation, cell viability was assessed by Calcein‐AM/PI live‐dead staining and MTT assays. A significant increase in dead cells and a marked reduction in cell viability confirmed that RC@NMVs effectively induced calcium overload‐driven cytotoxicity (Figure 2H–K). Consistent with these findings, similar concentration‐dependent cytotoxicity was also observed in other murine cancer cell lines, including 4T1 (breast cancer) and B16 (melanoma), further demonstrating the broad therapeutic potential of RC@NMVs across different tumor types (Figure S4). Notably, within this concentration range, free RR alone did not exhibit significant cytotoxic effects against either 4T1 or B16 cells. This finding further indicates that the observed cytotoxicity of RC@NMVs does not stem from any single component, but rather depends on the co‑delivery and synergistic interaction between RR and calcium ions (Figure S5). Overall, these results demonstrate that RC@NMVs effectively induce intracellular calcium overload through a synergistic mechanism, thereby triggering potent tumor cell cytotoxicity (Figure 2L).

### 2.3 Calcium Overload‐Induced Pyroptosis and Immune Activation by RC@NMVs

**FIGURE 3:**
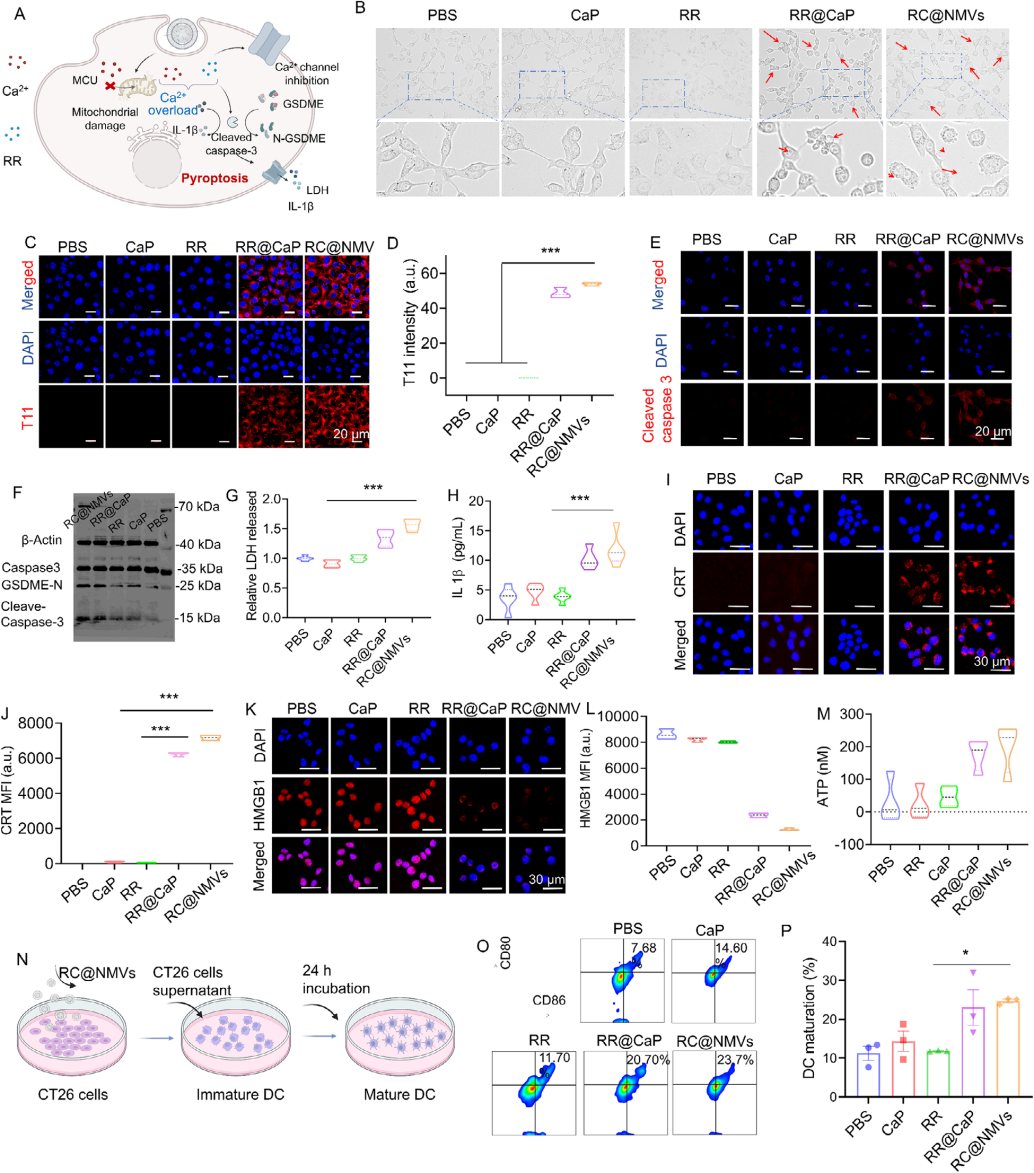
RC@NMVs‐induced calcium overload triggers pyroptosis and promotes immune activation. (A) Schematic illustration showing RC@NMVs‐mediated intracellular calcium overload triggering pyroptosis in tumor cells. (B) Representative bright‐field images showing morphological changes in CT26 cells after different treatments. (C,D). Confocal fluorescence images (C) and corresponding quantitative analysis (D) of CT26 cells stained with T11 dye, indicating membrane integrity disruption. (E) Confocal fluorescence images of cleaved caspase‐3 expression in CT26 cells. (F) Western blot analysis of pyroptosis‐related proteins in CT26 cells with different treatments. (G) The LDH release from CT26 cells with different treatments. (H) ELISA analysis of IL‐1β secretion from CT26 cells with different treatments. (I,J) Confocal fluorescence images (I) and corresponding quantitative analysis (J) showing the surface exposure of CRT on CT26 cells after different treatments. (K,L) Confocal fluorescence images (K) and corresponding quantitative analysis (L) showing the HMGB1 release from CT26 cells after different treatments. (M) Quantification of extracellular ATP release from CT26 cells after different treatments. (N) Schematic diagram illustrating the maturation of dendritic cells induced by DAMPs released from pyroptotic tumor cells pre‐treated by RC@NMVs. (O) Flow cytometry analysis of CD11c^+^ dendritic cells expressing co‐stimulatory markers CD80 and CD86 after incubation with the supernatants of CT26 cells subjected to different treatments. (P) Quantification of mature dendritic cells (CD11c^+^, CD80^+^, and CD86^+^) from flow cytometry data in (O). Data are expressed as mean ± SEM. Statistical significance was analyzed using one‐way ANOVA with Tukey's multiple comparison test; ^*^ *p* < 0.05, ^**^ *p* < 0.01, ^***^ *p* <0.001.

Calcium ion stress has been reported to induce pyroptosis, a form of inflammatory cell death [30, 31]. To investigate the effect of RC@NMVs‐medicated calcium overload in triggering cell pyroptosis (Figure 3A), we first examined the morphological characteristics of pyroptosis by microscopy. As shown in Figure 3B, Cells treated with RC@NMVs or RC exhibited typical pyroptotic features, including membrane blebbing and cell swelling, with small protrusions on the cell surface, indicating membrane rupture. Whereas, cells treated with RR or CaP nanoparticles displayed no significant morphological changes. Additionally, we used the T11 dye, which cannot penetrate an intact cell membrane, to assess pyroptosis. In RC@NMVs or RR‐treated cells, enhanced intracellular fluorescence was observed, indicating increased cell membrane permeability, a hallmark of pyroptotic cell death (Figure 3C,D). To further confirm the induction of pyroptosis, we assessed the activation of caspase‐3, a key marker of pyroptotic signaling, through confocal microscopy. We observed that the cleaved‐caspase 3 fluorescence intensity was notably increased in RR or RC@NMVs‐treated cells (Figure 3E). Western blot analysis corroborated the above results, showing a significant increase in cleaved‐caspase 3 and GSDME‐N, both of which are associated with pyroptosis (Figure 3F). Finally, we measured the release of lactate dehydrogenase (LDH) and interleukin‐1β (IL‐1β), as these are indicative of cell membrane rupture and the initiation of an inflammatory response, respectively. In RC or RC@NMVs‐treated cells, significant increases in both LDH and IL‐1β release were observed, further confirming the induction of pyroptosis and the subsequent immune activation (Figure 3G,H).

Moreover, we further investigated additional cell death pathways activated by RC@NMVs. As shown in Figure S6, treatment with RC@NMVs resulted in a substantial increase in apoptotic cell populations, as quantified by annexin V‐FITC/PI flow cytometry. Furthermore, BODIPY^TM^ 581/591 C11 staining confirmed that RC@NMVs treatment most potently induced lipid peroxidation, a hallmark of ferroptosis (Figure S7). These results demonstrate that RC@NMVs concurrently activate pyroptotic, apoptotic, and ferroptotic pathways, thereby driving a robust multimodal cell death response.

Next, we investigated the potential of RC@NMVs to induce immunogenic cell death (ICD) through pyroptosis and stimulate antitumor immune responses. First, to evaluate the hallmark features of ICD, we assessed the cell surface translocation of calreticulin (CRT), the nuclear‐to‐cytoplasmic translocation of high mobility group box 1 (HMGB1), and extracellular ATP secretion. CLSM imaging revealed that RC or RC@NMVs‐treated cells exhibited markedly enhanced CRT exposure on the plasma membrane and substantial cytoplasmic release of HMGB1, compared to other treated groups (Figure 3I–L). In addition, RC or RC@NMVs treatments significantly promoted ATP secretion, indicative of robust ICD induction (Figure 3M). Subsequently, we evaluated dendritic cell (DC) maturation by measuring the expression of CD80 and CD86 on bone marrow‐derived dendritic cells co‐cultured with treated CT26 cells (Figure 3N). Flow cytometry analysis revealed that RC or RC@NMVs treatments significantly promoted DC maturation, with the proportion of matured DCs (CD11c^+^CD80^+^CD86^+^) reaching 23.0% and 24.7%, respectively, which was notably higher than that observed in the CaP (14.3%), or RR (11.8%) groups (Figure 3O,P).

Therefore, these results suggest that RC@NMVs not only induce pyroptotic cell death but also trigger potent ICD and immune activation.

### 2.4 Inflammation‐Amplified Tumor Targeting Mediated by RC@NMVs

**FIGURE 4:**
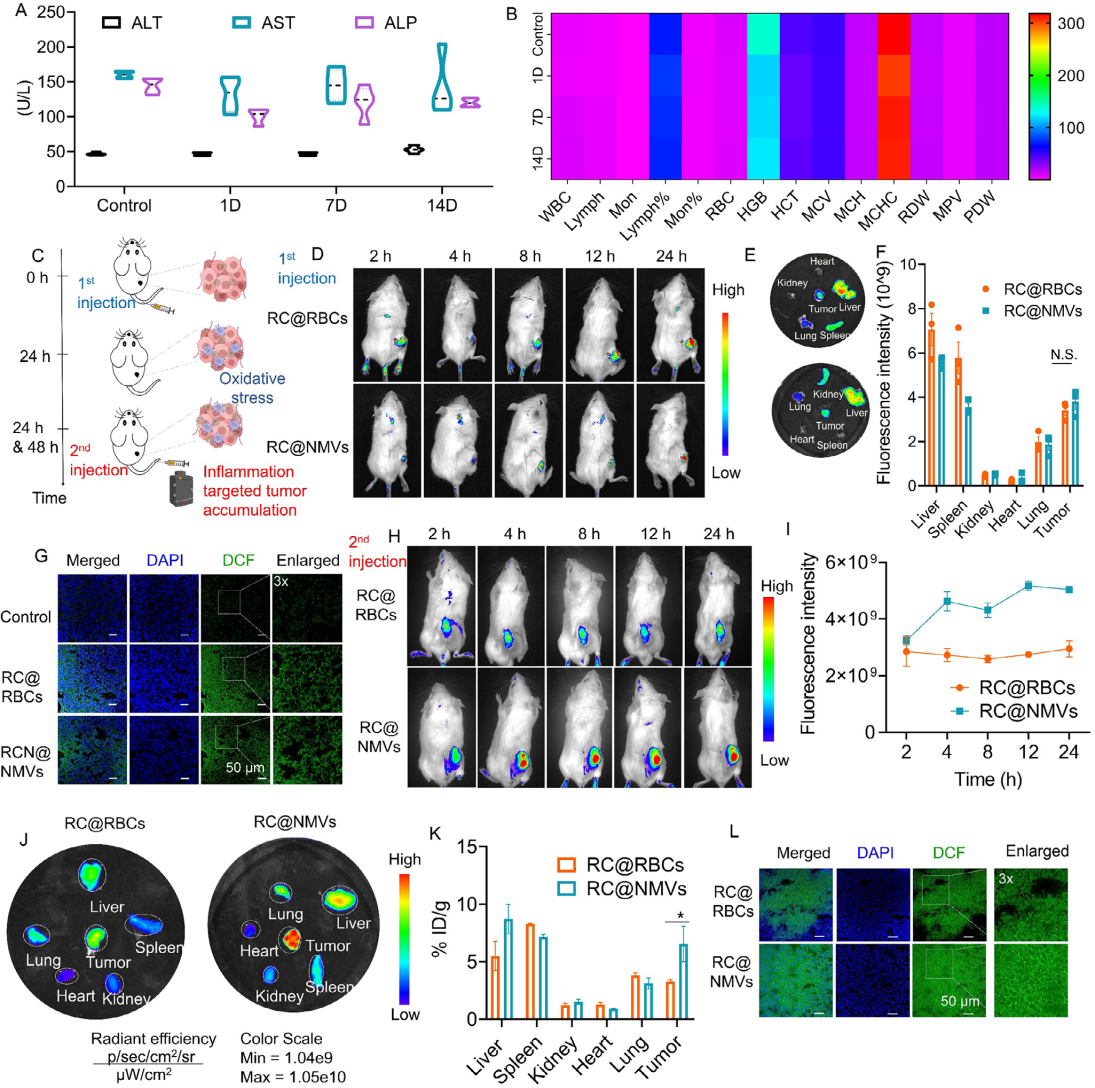
RC@NMVs‐mediated inflammation amplification for enhancing tumor accumulation. (A) Blood biochemistry analysis of mice after treatment with RC@NMVs, including ALT, AST, and ALP. (B) Complete blood count analysis of mice after treatment with RC@NMVs. (C) Schematic illustration of the experimental design. (D) In vivo fluorescence imaging of CT26 tumor‐bearing mice at different time points post‐injection of DiD‐labeled RC@NMVs and RC@RBCs (n = 3). Red blood cell membrane‐coated nanoparticles (RC@RBCs) were used as a control group. (E,F) Ex vivo fluorescence imaging (E) and corresponding quantitative analysis (F) of major organs and tumors at 24 h post‐injection. (G) Representative fluorescence images of tumor sections stained for ROS at 24 h post‐injection of RC@NMVs or RC@RBCs. (H,I) In vivo fluorescence imaging (H) and corresponding quantitative analysis (I) of CT26 tumor‐bearing mice were performed at different time points following a second injection of DiD‐labeled RC@NMVs or RC@RBCs, which was administered 24 h after an initial non‐labeled nanoparticle injection (n = 3). (J) Ex vivo fluorescence imaging of major organs and tumors at 24 h post the second injection. (K) Biodistribution profiles of RC@NMVs and RC@RBCs in major organs and tumors at 24 h after the second injection, expressed as percentage of injected dose per gram of tissue (%ID/g). (L) Representative fluorescence images of tumor sections stained for ROS at 24 h after the second injection of RC@NMVs or RC@RBCs. Data are expressed as mean ± SEM. Statistical significance in F, K was analyzed using an unpaired two‐tailed Student's *t*‐test; ^*^ *p* < 0.05, ^**^ *p* < 0.01, ^***^ *p* <0.001; n.s. indicates no statistically significant difference.

Neutrophils are known for their inherent chemotactic migration toward inflamed tissues [7, 32]. Given that RC@NMVs‐mediated calcium overload could induce pyroptotic cell death, which might remodel the tumor microenvironment into a more inflamed and immunostimulatory state. This, in turn, may further enhance tumor accumulation of subsequently administered RC@NMVs via neutrophil membrane‐mediated inflammation targeting. To ensure biosafety, we first evaluated the systemic toxicity of RC@NMVs via blood biochemical and hematological analyses. As shown in Figure 4A,B, all evaluated parameters remained within the normal physiological range, indicating excellent biocompatibility of RC@NMVs. Moreover, serum levels of major pro‐inflammatory cytokines (TNF‐α, IL‐6, and IL‐1β) were monitored and showed no significant difference from the untreated group, further confirming the absence of a harmful systemic inflammatory response (Figure S8). We next assessed the tumor‐targeting capability and inflammation‐amplifying effect of RC@NMVs using a CT26 subcutaneous tumor model (Figure 4C). Red blood cell membrane‐coated nanoparticles (RC@RBCs) were used as a control. Via an IVIS optical imaging system, it was found that upon intravenous administration of DiD‐labeled RC@NMVs and RC@RBCs, both groups exhibited comparable tumor accumulation (Figure 4D–F). At 24 h after the first injection, tumor tissues were collected and sectioned for ROS staining, which revealed similarly elevated levels of intratumoral oxidative stress in both RC@NMVs‐ and RC@RBCs‐treated groups, significantly higher than that observed in the control group (Figure 4G). Strikingly, following a second injection administered 24 h later, RC@NMVs exhibited markedly enhanced tumor accumulation compared to RC@RBCs, as shown by in vivo and ex vivo tissue imaging (Figure 4H–J). Quantitative biodistribution analysis revealed a tumor accumulation of 6.5% ID/g for RC@NMVs, significantly higher than that of 3.3% ID/g for RC@RBCs (Figure 4K). Furthermore, ROS staining of tumor sections revealed more intense oxidative stress in the RC@NMVs group than in the RC@RBCs, indicating elevated local inflammation (Figure 4L). These results can be explained by the fact that the initial administration of RC@NMVs amplifies the inflammatory microenvironment within tumors, thereby facilitating subsequent neutrophil membrane‐mediated homing and enhanced tumor accumulation. In contrast, RC@RBCs lack this inflammation‐responsive targeting moiety. Thus, RC@NMVs effectively leverage inflammation amplification to significantly boost tumor‐targeting efficiency.

### 2.5 The Therapeutic Effect of RC@NMVs Against Tumor Progression and Metastasis Via Inflammation‐Amplified Targeting

**FIGURE 5:**
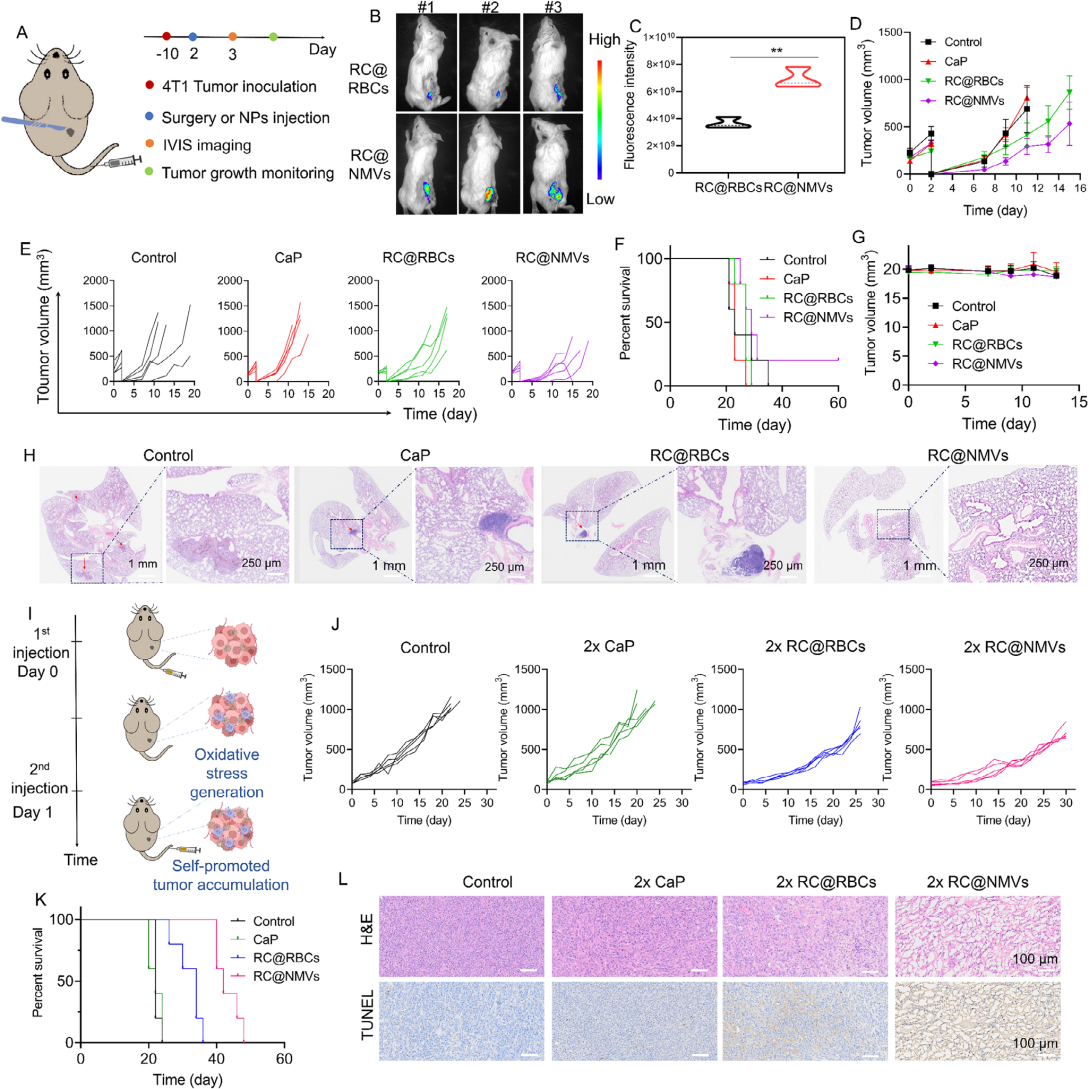
The therapeutic effect of RC@NMVs against tumor progression and metastasis via inflammation‐amplified targeting. (A) Schematic illustration of the experimental design for the postoperative tumor recurrence model. (B,C) In vivo fluorescence imaging (B) and corresponding quantitative analysis (C) of 4T1 tumor‐bearing mice that received intravenous injections of DiD‐labeled RC@NMVs or RC@RBCs following surgical resection of primary tumors, to assess nanoparticle accumulation at residual tumor sites. (D,E) Tumor recurrence growth curves of 4T1 tumor‐bearing mice after various treatments (n = 5). (F) Survival curves of mice with 4T1 tumor‐bearing after various treatments. (G) Body weight changes of 4T1 tumor‐bearing mice over the treatment period. (H) Representative histological images of lung sections from different treatment groups stained with H&E to evaluate pulmonary metastasis. (I) Schematic illustration of the experimental design for the self‐amplifying tumor enrichment and killing model via repeated injections of RC@NMVs. (J‐K) Individual tumor growth curves (J) and survival curves (K) following twice injections of various nanoformulations. (L) Representative histological images of lung sections from different treatment groups stained with H&E and TUNEL showing histological changes and apoptosis, respectively. Data are expressed as mean ± SEM. Statistical significance in C was analyzed using an unpaired two‐tailed Student's *t*‐test; ^*^ *p* < 0.05, ^**^ *p* < 0.01, ^***^ *p* <0.001.

Previous studies have reported that surgical resection of tumors often induces localized inflammation at the residual tumor site [33, 34]. To investigate whether RC@NMVs could exploit this inflammation for enhanced targeting, a subcutaneous 4T1 tumor model was established, followed by partial surgical resection of the primary tumor. Subsequently, mice were intravenously injected with equal doses of DiD‐labeled RC@NMVs or RC@RBCs (Figure 5A). In vivo fluorescence imaging revealed that the RC@NMVs group exhibited markedly higher tumor accumulation at the surgical site compared to RC@RBCs (Figure 5B,C). This enhancement is attributed to the inflammation‐responsive targeting ability of neutrophil membranes, which facilitates preferential homing to the inflamed tumor microenvironment. Then, we evaluated tumor recurrence and progression after surgery. Tumor growth curves revealed that a single injection of RC@NMVs effectively suppressed postoperative tumor regrowth compared to RC@RBCs, resulting in a delay in tumor recurrence and prolonged survival. In contrast, CaP alone exhibited negligible antitumor activity, showing similar tumor progression as the control group (Figure 5D–F). To further confirm the in vivo induction of pyroptosis by RC@NMVs, we performed immunofluorescence staining of tumor sections for specific pyroptosis markers. The analysis revealed a marked enhancement in the expression of Cleaved‐Caspase 3 and GSDME‐N in tumors treated with RC@NMVs compared to control groups, providing direct histological evidence that RC@NMVs effectively trigger pyroptosis within the tumor microenvironment (Figures S9 and S10). Throughout the monitoring period, no significant changes in body weight were observed among the different treatment groups, indicating good biocompatibility (Figure 5G). To further investigate the inhibitory effect of RC@NMVs on tumor metastasis, histological analysis of lung tissue sections was performed. Hematoxylin and eosin (H&E) staining revealed that the RC@NMVs group exhibited markedly fewer metastatic nodules in the lungs compared to RC@RBCs, suggesting that RC@NMVs not only suppress local tumor recurrence but also reduce distant metastasis, likely through inflammation‐amplified targeting and enhanced therapeutic efficacy (Figure 5H).

Then, to further validate the potential of RC@NMVs‐mediated inflammation‐amplified targeting for enhanced tumor accumulation and therapeutic efficacy, a subcutaneous 4T1 tumor model was re‐established, followed by two sequential injections of RC@NMVs at 24‐h intervals (Figure 5I). Tumor growth curves indicated that RC@NMVs achieved markedly superior tumor suppression and extended survival compared to RC@RBCs (Figure 5J; K, Figure S11). At 24 h after the second injection, tumor tissues were harvested for H&E and TUNEL staining. As shown in Figure 5L, RC@NMVs treatment resulted in the most extensive tumor necrosis and apoptosis among all groups, highlighting the enhanced antitumor potency achieved through inflammation amplification by RC@NMVs. Importantly, the RC@NMVs treatment caused no significant changes in body weight and elicited no detectable histopathological damage in major organs, including the heart, liver, spleen, lungs, and kidneys, demonstrating excellent biosafety (Figures S12; Figure S13).

### 2.6 Therapeutic and Immunostimulatory Effects of RC@NMVs in CT26 Tumors

**FIGURE 6:**
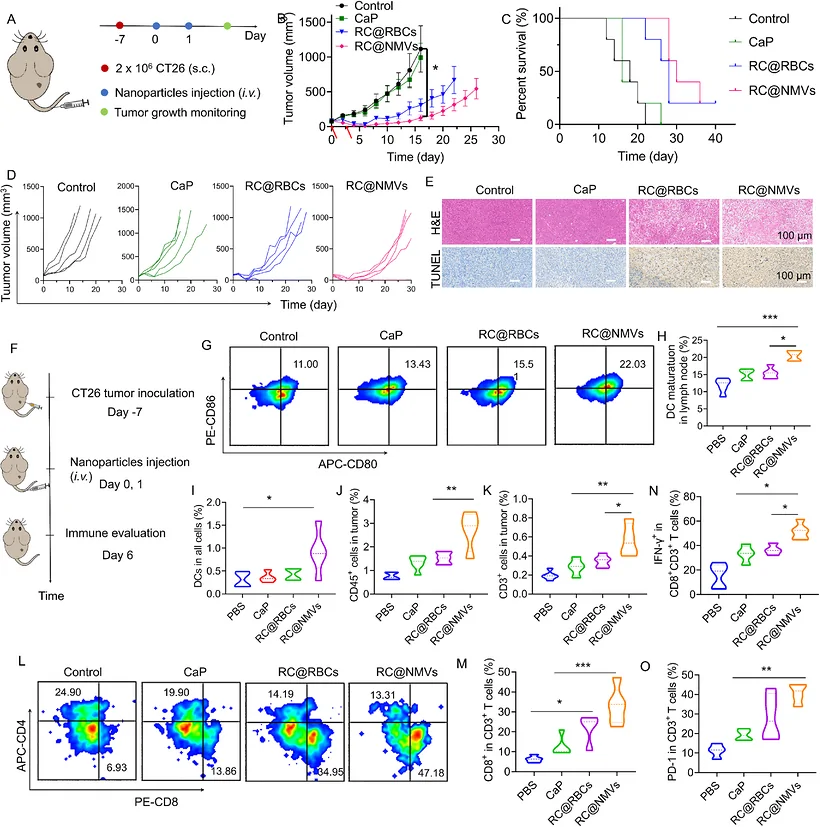
RC@NMVs‐Mediated Antitumor Efficacy and Immune Activation in a CT26 Model. (A) Schematic illustration of the treatment schedule. (B,C) Average tumor growth curves (B) and individual tumor growth curves (C) of B16F10 tumor‐bearing mice with different treatments (n = 5). (D) Survival curves of B16F10 tumor‐bearing mice receiving various treatments. (E) Representative images of tumor sections stained with H&E and TUNEL. (F) Schematic illustration of the immune evaluation schedule. (G,H) Typical flow cytometry plots (G) and quantification (H) showing the maturation of dendritic cells (DCs, CD11c^+^, CD86^+^, and CD80^+^) in tumor‐draining lymph nodes (TDLNs) after different treatments (n = 5). (I) Quantification of DC infiltration (CD11c^+^) within tumor tissues after different treatments. (J,L) Flow cytometry analysis of the proportion of tumor‐infiltrating leukocytes (CD5^+^) (J) and CD3^+^ T cells (K) after different treatments. (L,M) Flow cytometry plots (L) and quantification (M) of intratumoral CD8^+^ T cell infiltration after different treatments. (N) Flow cytometry analysis of the proportion of IFN‐γ in CD8^+^CD3^+^ T cells after different treatments. (O) Expression levels of PD‐1 on tumor‐infiltrating T cells after different treatments, shown as the proportion of PD‐1^+^ cells among CD3^+^ T cells. Data are expressed as mean ± SEM. Statistical significance was analyzed using one‐way ANOVA with Tukey's multiple comparison test; ^*^ *p* < 0.05, ^**^ *p* < 0.01, ^***^ *p* <0.001.

Subsequently, we evaluated the therapeutic efficacy and immune activation of RC@NMVs in a subcutaneous CT26 colon cancer model. Following the established therapeutic schedule, CT26 tumor‐bearing mice received two intravenous doses of RC@NMVs (on days 0 and 1), and tumor progression was subsequently monitored to evaluate therapeutic efficacy (Figure 6A). As expected, it was found that dual administration of RC@NMVs resulted in the most effective tumor suppression and significantly prolonged survival (Figure 6B–D). Histological analysis, including H&E and TUNEL staining of tumor sections, further confirmed the strong antitumor effect of RC@NMVs, showing extensive tumor cell apoptosis and necrosis (Figure 6E). Moreover, histological analysis of major organs revealed no discernible pathological changes following RC@NMVs treatment (Figure S14). These results collectively suggest that RC@NMVs not only effectively inhibit tumor progression but also elicit a potent therapeutic response with minimal systemic toxicity.

Furthermore, we systematically investigated the underlying immunological mechanisms responsible for the RC@NMVs‐mediated tumor suppression. Following the treatment protocol, mice received twice intravenous injections of RC@NMVs, and tumor tissues along with the adjacent draining lymph nodes (dLNs) were harvested on day 6 post‐treatment for immune evaluation (Figure 6F). We first assessed dendritic cell (DC) maturation within the dLNs. Flow cytometry analysis revealed that the proportion of mature DCs (CD80^+^CD86^+^) significantly increased from 11.9% in the control group to 20.6% following RC@NMVs treatment, indicating enhanced antigen presentation and initiation of antitumor immunity (Figure 6G,H). Similar trends were observed in the tumor microenvironment, where both the proportion of intratumoral DCs (CD11c^+^) and their maturation status (CD80^+^, CD86^+^) markedly increased following RC@NMVs treatment (Figure 6I; Figure S15). Subsequent analysis of immune cell infiltration revealed a robust increase in total leukocytes (CD45^+^) and T cells (CD3^+^) within the tumor microenvironment (Figure 6J,K; Figure S16). Notably, the proportion of cytotoxic CD8^+^ T cells markedly increased from ∼6.31% in the control group to 33.0% following RC@NMVs treatment, indicating enhanced effector cell recruitment (Figure 6L,M). Moreover, the ratio of CD8^+^ to Regulatory T cells exhibited a significant elevation, suggesting a shift in the tumor microenvironment toward immune activation and suppressed immunosuppressive regulation (Figure S17). Furthermore, the population of IFN‐γ^+^CD8^+^ T cells, indicative of functional activation, also rose significantly, highlighting the potent cytotoxic immune response elicited by the treatment (Figure 6N; Figure S18). In addition, we examined the impact of RC@NMVs treatment on immune checkpoint expression. Flow cytometry analysis revealed a notable increase in the proportion of PD‐1^+^ cells among CD3^+^ T cells, suggesting heightened T cell activation accompanied by potential exhaustion (Figure 6O; Figure S19).

Collectively, these results demonstrate that RC@NMVs not only induce pyroptosis‐mediated tumor inflammation but also promote robust dendritic cell activation and T cell‐mediated adaptive immunity, thereby contributing to durable antitumor effects.

### 2.7 Abscopal Antitumor Effect Induced by RC@NMVs Combined with Immune Checkpoint Blockade Therapy

**FIGURE 7:**
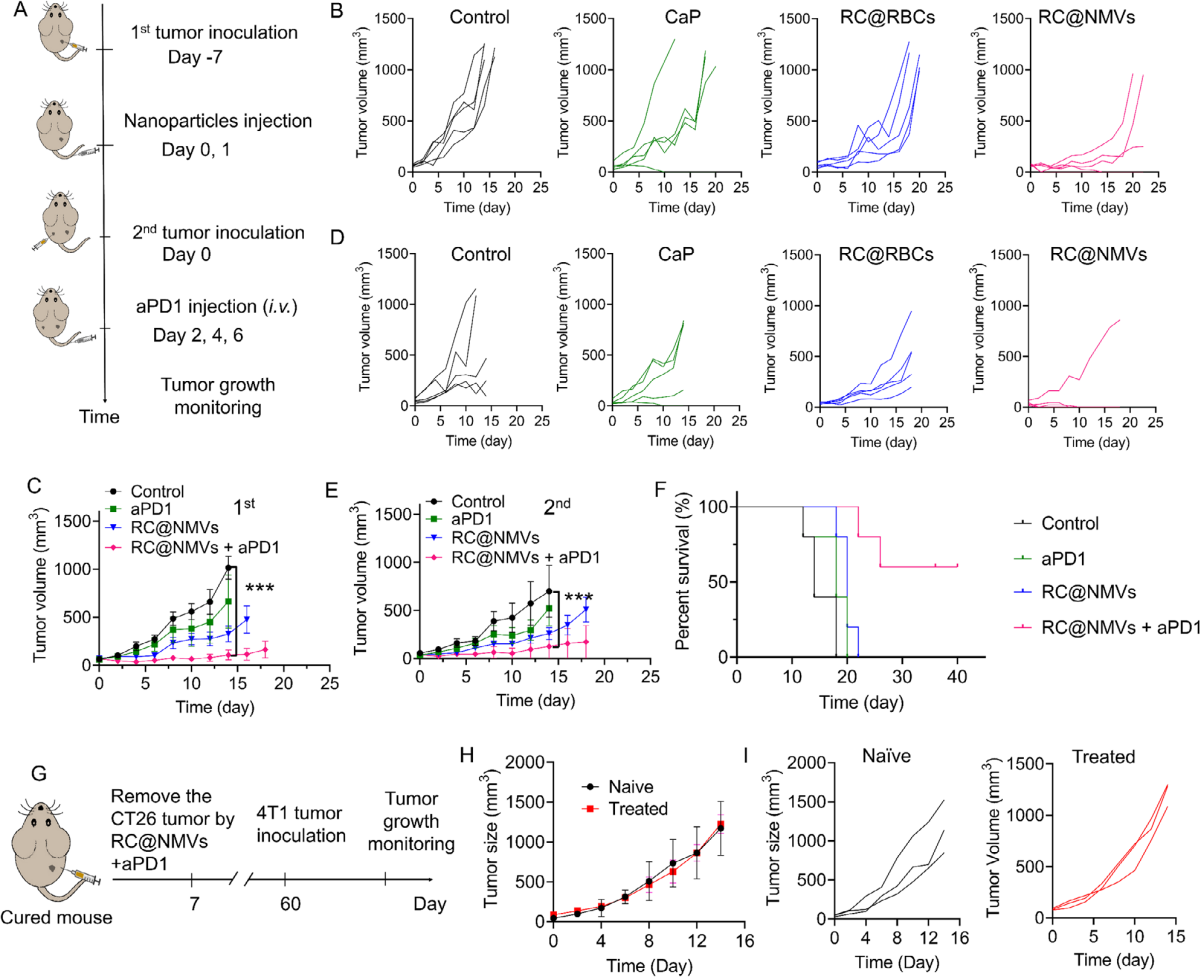
Abscopal antitumor effect induced by RC@NMVs combined with immune checkpoint blockade therapy. (A) Schematic illustration of the treatment schedule in the bilateral CT26 tumor model. (B,C) The individual tumor growth (B) and average tumor growth curves (C) of the primary tumor after different treatments (n = 5). (D,E) The individual tumor growth (D) and average tumor growth curves (E) of the distant tumor after different treatments. (F) Survival curves of mice bearing bilateral CT26 tumors after different treatments. (G) Body weight changes of mice during the monitoring period after various treatments. (H) Schematic illustration of the tumor rechallenge experiment, in which the cured mice were rechallenged with 4T1 tumor cells on day 60 to evaluate antigen‐specific immune memory. (H,I) Tumor growth curves of rechallenged mice (H) and individual tumor growth curves (I), with naïve mice used as controls. Data are expressed as mean ± SEM. Statistical significance in C, E was analyzed using an unpaired two‐tailed Student's *t*‐test; ^*^ *p* < 0.05, ^**^ *p* < 0.01, ^***^ *p* <0.001.

Given that RC@NMVs treatment upregulated PD‐1 expression on CD3^+^ T cells, this observation highlights the potential benefit of combining RC@NMVs with immune checkpoint blockade to further augment antitumor efficacy. To validate this hypothesis, we established a bilateral subcutaneous CT26 tumor model in mice, where one tumor served as the primary treatment site and the contralateral tumor represented distant lesions. Mice received intravenous injections of RC@NMVs on days 0 and 1, followed by intravenous administrations of anti‐PD‐1 antibodies on days 2, 4, and 6, according to a designated treatment schedule (Figure 7A). Excitingly, the combination therapy significantly suppressed the growth of both primary and distant tumors compared to either anti‐PD‐1 antibodies or RC@NMVs therapy (Figure 7B–E). Remarkably, complete tumor regression was achieved in 3 out of 5 mice, indicating the induction of a potent systemic antitumor immune response (Figure 7F). Throughout the monitoring period, no significant body weight loss was observed, suggesting favorable biosafety (Figure S20). To evaluate the induction of antigen‐specific immune memory, cured mice were rechallenged with a different tumor type (4T1), with naïve mice serving as controls (Figure 7G). As shown by the tumor growth curves, no significant difference in tumor growth was observed between the two groups, indicating that the protective immune response elicited by RC@NMVs treatment was antigen‐specific and did not confer cross‐protection against unrelated tumor types (Figure 7H,I).

Therefore, these results verify the synergistic potential of inflammation‐amplifying nanotherapy with immune checkpoint inhibition, offering a promising strategy for converting immunologically “cold” tumors into “hot” ones and achieving durable therapeutic outcomes.

## 3 Conclusion

In summary, we developed an inflammation‐amplifying tumor‐targeting strategy that effectively reverses the immunosuppressive features of cold tumors through calcium overload‐enhanced pyroptosis and immune activation. Upon tumor cell internalization, RC@NMVs released Ca^2^^+^ and ruthenium red to synergistically disrupt calcium homeostasis, triggering gasdermin‐mediated pyroptotic cell death. The resulting release of DAMPs and pro‐inflammatory cytokines amplified the local inflammatory microenvironment, which in turn facilitated the inflammation‐directed recruitment and tumor enrichment of subsequently administered RC@NMVs via neutrophil membrane‐mediated chemotaxis. This self‐reinforcing accumulation strategy substantially enhanced the overall therapeutic efficacy and immune activation. We demonstrated that RC@NMVs induced robust pyroptosis, significantly inhibited tumor growth, and prolonged survival. Importantly, the pyroptosis‐driven inflammation elicited durable systemic antitumor immune responses, preventing tumor recurrence and metastasis. Furthermore, combining RC@NMVs with immune checkpoint blockade therapy led to markedly improved therapeutic outcomes. Altogether, this work presents a promising strategy that couples inflammation‐mediated targeting with pyroptosis amplification to boost immune responses and improve the treatment of immunologically “cold” tumors.

## 4 Experimental Section

### 4.1 Materials

Calcium chloride dihydrate (CaCl_2_·2H_2_O), and disodium hydrogen phosphate (Na_2_HPO_4_) were purchased from Sinopharm Chemical Reagent Co., Ltd., China. Poly(γ‐glutamic acid)‐b‐poly(ethylene glycol) (PGA_5K_‐PEG_10K_) was obtained from Xi'an Ruixi Biological Technology Co., Ltd., China. Ruthenium red was purchased from MeilunBio Co., Ltd., China. DiD dye, Fluo‐4 AM, mitochondrial membrane potential assay kit (JC‐1), and 2′,7′‐dichlorofluorescin diacetate (DCFH‐DA)were purchased from Beyotime Biotechnology Co., Ltd., China. Annexin V‐FITC/PI assay kits were sourced from YiFeiXue Biotechnology Co., Ltd., China. BODIPY^TM^ 581/591 C11 was obtained from Beyotime Co., Ltd., China. Antibodies against cleaved caspase‐3, GSDME‐N, HMGB1, and CRT were obtained from Abcam. Anti‐PD‐1 antibody was obtained from BioXcell. Antibodies for flow cytometry assays were obtained from BioLegend or Invitrogen.

### 4.2 Synthesis of CaP and RR@CaP Nanoparticles

CaP nanoparticles were synthesized via a water‐phase co‐precipitation method using PGA_5k_‐PEG_10k_ as a stabilizing template [35]. Briefly, aqueous solutions of CaCl_2_·2H_2_O and Na_2_HPO_4_ were rapidly mixed under vigorous stirring in the presence of PGA_5k_‐PEG_10k_, resulting in the formation of uniform CaP nanoparticles. The obtained CaP nanoparticles were collected by centrifugation at 10 000 rpm for 5 min and washed with deionized water.

For ruthenium red loading, different amounts of ruthenium red were added to an aqueous solution containing CaP nanoparticles and stirred at room temperature for 12 h. Then, the mixture was centrifuged at 10 000 rpm for 5 min to remove unbound ruthenium red. The drug loading content and encapsulation efficiency were determined by measuring the absorbance of ruthenium red at 450 nm using UV–vis spectroscopy.

### 4.3 Preparation of Neutrophil Membrane Vesicles

Neutrophils were freshly isolated from the bone marrow of healthy BALB/c mice using density gradient centrifugation. Cell membrane vesicles were prepared through repeated freeze‐thaw cycles and subsequent mechanical disruption. The resulting neutrophil membrane fragments were subjected to differential centrifugation to remove nuclear and mitochondrial debris, followed by extrusion through a 200 nm polycarbonate membrane to obtain uniform neutrophil membrane vesicles.

### 4.4 Fabrication of RC@NMVs Nanoparticles

To fabricate neutrophil membrane‐coated RR@CaP nanoparticles (RC@NMVs), purified NMVs and RR@CaP cores were first mixed at a 1:10 mass ratio, followed by mild sonication to promote initial interaction. The mixture was then extruded through a 200 nm polycarbonate membrane using a mini‐extruder, facilitating membrane fusion and yielding biomimetic RC@NMVs. SDS‐PAGE analysis was performed to verify the successful coating of RR@CaP nanoparticles with neutrophil membranes.

### 4.5 Characterization

The morphology and elemental distribution of RC@NMVs were analyzed by transmission electron microscopy (TEM, Talos F200i, Sigma) equipped with energy‐dispersive x‐ray spectroscopy. The elemental composition was further confirmed by x‐ray photoelectron spectroscopy (XPS, Thermo Kalpha). The hydrodynamic diameter and zeta potential were measured using a Malvern Zetasizer (Zetasizer Pro). The successful coating of neutrophil membranes onto RR@CaP nanoparticles was verified by SDS‐PAGE analysis. The loading content of ruthenium red was quantified via UV–vis spectroscopy based on its specific absorbance (GENESYS50, Thermo Fisher).

### 4.6 pH‐Responsive Release Behavior of RC@NMVs

To evaluate the pH‐responsive Ca^2+^ ions and ruthenium red release, RC@NMVs were incubated in PBS buffers with different pH values (5.5, 6.5, and 7.4) at 37 °C. At predetermined time points, the suspensions were centrifuged at 10 000 rpm for 3 min to collect the supernatant. The released Ca^2^^+^ and ruthenium red in the supernatant were quantified by ICP‐MS and UV–vis spectroscopy, respectively.

### 4.7 Cell Experiments

The mouse mammary carcinoma cell line 4T1  (RRID: CVCL_0125), colon carcinoma cell line CT26 (RRID: CVCL_7254), and melanoma cell line B16F10 (RRID: CVCL_0159) were purchased from the Cell Bank of the Shanghai Institutes for Biological Sciences, Chinese Academy of Sciences. All cell lines were authenticated and routinely tested to be free of mycoplasma contamination. 4T1 and CT26 cells were maintained in RPMI 1640 medium, while B16F10 cells were cultured in Dulbecco's Modified Eagle Medium (DMEM). All media were supplemented with 10% fetal bovine serum (FBS) and 1% penicillin‐streptomycin, and the cells were incubated at 37°C in a humidified atmosphere with 5% CO_2_.

To assess intracellular Ca^2^^+^ overload induced by RC@NMVs, CT26 cells were seeded in confocal dishes at a density of 2 × 10^5^ cells per well and incubated overnight. After treatment with RR, CaP, RR@CaP, and RC@NMVs for 4 h, the cells were washed and incubated with 5 µM Fluo‐4 AM dye for 30 min at 37 °C in the dark. Cells were then washed three times with PBS and observed using a CLSM.

To evaluate intracellular ROS generation, cells were incubated with RR, CaP, RR@CaP, and RC@NMVs for 6 h, followed by staining with 10 µM DCFH‐DA for 30 min at 37 °C. After removing excess dye with PBS, green fluorescence from oxidized DCF was visualized by CLSM.

To determine mitochondrial dysfunction, cells were treated with RR, CaP, RR@CaP, and RC@NMVs and then incubated with JC‐1 staining solution for 20 min at 37 °C. Cells were rinsed with PBS and imaged by CLSM. Healthy mitochondria showed red fluorescence (JC‐1 aggregates), while depolarized mitochondria emitted green fluorescence (JC‐1 monomers).

Cell morphology was directly observed under CLSM following RR, CaP, RR@CaP, and RC@NMVs treatments. For pyroptosis detection, those treated cells were stained with 4‐(Dimethylamino) benzene‐1,3‐diol (T11) dye for 30 min at 37 °C in the dark. After washing, the cell nuclei were stained with DAPI before CLSM imaging.

To immunostain for the cleaved caspase‐3, cells were incubated with RR, CaP, RR@CaP, and RC@NMVs for 24 h. Then, the cells were fixed with 4% paraformaldehyde, permeabilized with 0.1% Triton X‐100, and incubated with anti‐cleaved caspase‐3 antibody overnight at 4 °C. After washing, the cells were treated with Alexa Fluor 594‐labeled secondary antibody for 1 h, followed by imaging with CLSM.

To investigate the immunogenic cell death induced by RC@NMVs, the translocation of HMGB1 and the exposure of CRT were assessed. For HMGB1 detection, cells were incubated with RR, CaP, RR@CaP, and RC@NMVs for 24 h. After treatments, the cells were incubated with anti‐HMGB1 antibody overnight at 4 °C, followed by Alexa Fluor 594‐labeled secondary antibody. For CRT staining, cells were fixed without permeabilization and incubated with anti‐CRT antibody, followed by Alexa Fluor 488‐conjugated secondary antibody. Nuclei were stained with DAPI, and images were acquired by CLSM.

ATP release from treated tumor cells was quantified to assess ICD. After incubation with RR, CaP, RR@CaP, and RC@NMVs for 24 h, the culture supernatants were collected. The ATP levels in the supernatant were measured using an ATP assay kit according to the manufacturer's instructions.

To evaluate DC maturation, BMDCs were co‐cultured with CT26 cells that had been pretreated with RR, CaP, RR@CaP, and RC@NMVs for 24 h. Subsequently, BMDCs were harvested and stained with fluorophore‐conjugated anti‐CD11c, anti‐CD80, and anti‐CD86 antibodies. After washing, cells were analyzed by flow cytometry.

### 4.8 Animal Experiments

All animal experiments were conducted in compliance with the guidelines approved by the Animal Welfare and Research Ethics Committee of Shandong First Medical University (Approval No. W202303030131). Female BALB/c mice (6‐8 weeks old) were purchased from Jinan Xingkang Biotechnology Co., Ltd. and maintained under specific pathogen‐free (SPF) conditions.

Inflammation‐amplified tumor targeting of RC@NMVs:

To evaluate the inflammation‐amplified tumor‐targeting capability of RC@NMVs, female BALB/c mice bearing subcutaneous CT26 tumors were intravenously injected with DiD‐labeled RC@NMVs or RC@RBCs at the equivalent concentration of 0.5 mg/kg. Fluorescence images were acquired at 2, 4, 8, 12, and 24 h post‐injection using an IVIS optical imaging system (PerkinElmer) to monitor real‐time biodistribution and tumor accumulation. At 24 h, mice were euthanized, and major organs (heart, liver, spleen, lung, kidney) as well as tumors were harvested for ex vivo fluorescence imaging. Tumor tissues were also subjected to ROS staining on cryosections to evaluate oxidative stress levels.

In addition, tumor‐bearing mice received two sequential intravenous injections to assess inflammation‐mediated targeting enhancement. In the first injection, RC@NMVs or RC@RBCs without DiD labeling were administered at an equivalent dose of 0.5 mg/kg. After 24 h, the mice received a second injection of DiD‐labeled RC@NMVs or RC@RBCs at the same dose. Fluorescence signals were then monitored at various time points using the IVIS optical imaging system. At 24 h post‐second injection, the mice were euthanized for ex vivo imaging of major organs and tumors. Tumor tissues were also collected and subjected to ROS staining on cryosections to evaluate the inflammation‐amplified tumor‐targeting capability.

In vivo antitumor effect of RC@NMVs:

To evaluate the in vivo antitumor efficacy of RC@NMVs, female BALB/c mice bearing subcutaneous 4T1 tumors were used in both postoperative recurrence and primary tumor models. The 4T1 tumor‐bearing mice model was established by subcutaneously inoculating 2 × 10^6^ 4T1 cells into the right flank. For the postoperative recurrence model, once the tumors reached ∼300 mm^3^, ∼95% of the tumor mass was surgically removed. Firstly, to establish the postoperative model, 4T1 tumors were allowed to grow to ∼300 mm^3^, followed by surgical removal of ∼95% of the tumor under anesthesia. At 24 h post‐surgery, mice were intravenously injected with DiD‐labeled RC@NMVs or RC@RBCs (0.5 mg/kg) (n = 3 per group). Fluorescence imaging was performed using an IVIS optical imaging system to assess tumor targeting in the postoperative site.

In the meanwhile, another set of postoperative 4T1 tumor‐bearing mice was treated to assess recurrence inhibition. Mice received intravenous injections of PBS, CaP, RC@RBCs, or RC@NMVs at 24 h post‐surgery (n = 5 per group). Tumor recurrence was monitored by measuring tumor volume with a digital caliper every other day, and body weights were recorded concurrently to evaluate systemic toxicity. Tumor volume (V) was calculated using the formula: V = L x W^2^/2, where L and W represent the tumor length and width, respectively. At the end of the observation period, lung tissues were harvested for H&E staining to evaluate pulmonary metastases.

To further evaluate the antitumor effect in a non‐surgical model, subcutaneous 4T1 tumor‐bearing mice (∼100 mm^3^) were treated with two intravenous injections of PBS, CaP, RC@RBCs, or RC@NMVs at a 24 h interval. The tumor volume and body weight of mice were monitored every other day. In addition, 24 h after the second injection, tumors were excised from mice for H&E and TUNEL staining to evaluate histological changes and apoptosis level, respectively. Furthermore, on day 14 post‐treatment, the major organs (heart, liver, spleen, lung, and kidney) from RC@NMVs‐treated and untreated mice were harvested for H&E staining.

To evaluate the in vivo antitumor efficacy of RC@NMVs in a CT26 subcutaneous tumor model, BALB/c mice were subcutaneously inoculated with 2 × 10^6^ CT26 cells into the right flank. Once tumors reached ∼80.1 mm^3^, mice were randomly divided into four groups (n = 5 per group) and intravenously administered with PBS, CaP, RC@RBCs, or RC@NMVs at an equivalent dosage of 0.5 mg/kg on days 0 and 1. Then, the tumor growth and body weight were recorded every other day.

For immunological evaluation, an additional batch of CT26 tumor‐bearing mice was established and treated under the same conditions as the above therapeutic study. On day 6 after the second injection, tumor‐draining lymph nodes and tumor tissues were collected for immunological analysis. Then, the collected tissues were processed into single‐cell suspensions following the standard protocols. For flow cytometry analysis, cells were stained with the following fluorescently conjugated antibodies, including including anti‐PerCP‐CD11b (clone M1/70, catalog: 101229), anti‐CD11c‐FITC (clone N418, catalog: 117306), anti‐CD80‐APC (clone 16‐10A1, catalog: 104714), anti‐CD86‐PE (clone GL‐1, catalog: 105008), anti‐CD45‐PerCP (clone 30‐F11, catalog: 103130), anti‐CD3‐FITC (clone 17A2, catalog: 100204), anti‐APC‐CD4 (clone GK1.5, catalog: 100412), anti‐CD8‐PE (clone 53–6.7, catalog: 100708), anti‐IFN‐γ‐PE‐Cy7 (clone XMG1.2, catalog: 505826), and anti‐PD‐1‐BV421 (clone 29F.1A12, catalog: 135221).

To evaluate the therapeutic effect of RC@NMVs combined with immune checkpoint blockade therapy, a bilateral CT26 tumor‐bearing mouse model was established. Female BALB/c mice were subcutaneously inoculated with 2 × 10^6^ CT26 cells into the right and left flanks at day ‐7 and ‐3, respectively. Once the primary tumor reached ∼100 mm^3^, the mice were randomly divided into four groups (n = 5 per group): PBS, aPD‐1, RC@NMVs, and RC@NMVs + aPD‐1. On day 0 and day 1, mice in the RC@NMVs and RC@NMVs + aPD‐1 groups were intravenously injected with RC@NMVs at a dosage of 0.5 mg/kg. On days 2, 4, and 6, the aPD‐1 antibody was administered intravenously at a dosage of 20 µg per mouse in the aPD‐1 and RC@NMVs + aPD‐1 groups. Tumor volume and body weight were monitored every other day.

Additionally, to verify antigen specificity, mice cured with RC@NMVs + aPD‐1 treatment were re‐challenged 40 days after the initial treatment. These cured mice were subcutaneously inoculated with 2 × 10^6^ 4T1 tumor cells on the right flank. Tumor volume and body weight were monitored every other day to assess the response to the new tumor challenge.

### 4.9 Statistical Analysis

All experiments were independently replicated at least three times. Data are presented as the mean ± standard error of the mean (SEM). The sample size (n) for each analysis represents the number of biologically independent replicates and is specified in the respective figure legends. Statistical comparisons between two groups were performed using an unpaired two‐tailed Student's *t*‐test. Comparisons among more than two groups were conducted using one‐way analysis of variance (ANOVA), followed by Tukey's multiple comparisons. All tests were two‐sided, and statistical significance was defined as ^*^ *p* < 0.05, ^**^ *p* < 0.01, and ^***^ *p* < 0.005; n.s. indicates no statistically significant difference. Data and statistical analysis were performed using GraphPad Prism software (version 8.5).

## Funding

National Natural Science Foundation of China (Nos. 52302350 and 22307057), the Shandong Provincial Natural Science Foundation (No. ZR2024QC370), the Natural Science Foundation of Jiangsu Province (No. BK20220401), the Shandong Provincial Taishan Scholars Program (tsqn202312237), and Qingfeng Program of Shandong First Medical University

## Conflicts of Interest

The authors declare no conflicts of interest.

## Supporting information




**Supporting File**: advs74933‐sup‐0001‐SuppMat.docx

## Data Availability

The data that support the findings of this study are available from the corresponding author upon reasonable request.;
