# Supplementary material for: Reinforcing Calcium Overload via Inflammation‐Mediated Targeting to Amplify Pyroptosis and Antitumor Immunity
Source: Adv Sci (Weinh). 2026 Mar 23;13(31):e22827. doi: 10.1002/advs.202522827 (PMC13587957; doi:10.1002/advs.202522827)
Supplement: Supplementary file 1 [file ADVS-13-e22827-s001.docx]

**Reinforcing Calcium Overload via Inflammation-Mediated Targeting to Amplify Pyroptosis and Antitumor Immunity**

Yingying Liu^1,2,4#^, Yang Liu^5#^, Qin Fan^3^*, Jinqiao Zhang^2^, Kexin Wen^2^, Boyu Yuan^2^, Xianzhou Cai^2^, Ziliang Dong^1,2^*, Lianhui Wang^3^*

1. Department of Clinical Pharmacy, The First Affiliated Hospital of Shandong First Medical University & Shandong Provincial Qianfoshan Hospital, Shandong Key Laboratory of Digital Diagnosis and Treatment of Thoracic Oncology, Jinan, Shandong, 250014, P. R. China.
2. Science and Technology Innovation Center, Shandong First Medical University& Shandong Academy of Medical Sciences, Jinan, Shandong, 250117, P. R. China.
3. State Key Laboratory of Flexible Electronics (LoFE) & Institute of Advanced Materials (IAM), Nanjing University of Posts & Telecommunications, Nanjing, Jiangsu, 210000, P. R. China.
4. Biomedical Sciences College, Shandong Medicinal Biotechnology Centre, Shandong First Medical University & Shandong Academy of Medical Sciences, Jinan, Shandong, 250117, P. R. China.
5. School of Chemistry, Chemical Engineering and Biotechnology, Nanyang Technological University, 21 Nanyang Link, Singapore 637371, Singapore.

E-mail: [iamqfan@njupt.edu.cn](mailto:iamqfan@njupt.edu.cn), [Zldong0220@163.com](mailto:Zldong0220@163.com), [iamlhwang@njupt.edu.cn](mailto:iamlhwang@njupt.edu.cn)

**Figure S1**. Typical TEM image of CaP nanoparticles.

**Figure S2.** Time-dependent size changes of RC@NMVs in 20% and 30% FBS, indicating their colloidal stability in physiological conditions (n = 3).

**Figure S3**. The Standard curve of Ruthenium red measured by UV-Vis spectrophotometry.

**Figure S4.** (A-B) Relative cell viability of 4T1 cells A) and B16 cells B) after incubation with different concentration of RC@NMVs**.**

**Figure S5.** (A-B) Relative cell viability of 4T1 cells A) and B16 cells B) after incubation with different concentration of Ruthenium Red**.**

**Figure S6.** (A, B) Flow cytometry analysis of apoptosis in cells treated with indicated formulations using Annexin V-FITC/PI staining A) and corresponding quantitative analysis B).

**Figure S7.** (A,B) Representative fluorescence images showing lipid peroxidation levels detected by BODIPY^TM^ 581/591 C11 staining (green: oxidized signal, red: reduced signal) A) and corresponding quantitative analysis B) to evaluate lipid peroxidation.

**Figure S8**. Systemic inflammatory cytokines in serum after RC@NMVs treatment. Levels of TNF-α A), IL-6 B), and IL-1β C) showed no significant difference (n.s.) from the untreated control group.

**Figure S9**. (A, B) Immunofluorescence staining A) and corresponding quantitative analysis B) of Cleaved-Caspase 3 in tumor sections from mice with different treatments. Statistical significance was analyzed using one-way ANOVA with Tukey’s multiple comparison test; * *P* < 0.05, ** *P* < 0.01, *** *P* <0.001.

**Figure S10**. (A, B) Immunofluorescence staining A) and corresponding quantitative analysis B) of GSDME-N in tumor sections from mice with different treatments. Statistical significance was analyzed using one-way ANOVA with Tukey’s multiple comparison test; * *P* < 0.05, ** *P* < 0.01, *** *P* <0.001.

**Figure S11**. Average tumor growth curves of 4T1 tumor-bearing mice with different treatments (n = 5).

**Figure S12**. The body weight of 4T1 tumor-bearing mice with different treatments (n = 3).

**Figure S13**. H&E-stained slices of major organs including heart, liver, spleen, lung and kidney of 4T1 tumor-bearing mice treated with RC@NMVs.

**Figure S14**. H&E-stained slices of major organs including heart, liver, spleen, lung and kidney of CT26 tumor-bearing mice treated with RC@NMVs.

**Figure S15**. Proportion of mature DCs in the tumor microenvironment after various treatments.

**Figure S16**. Typical flow cytometry plots of tumor-infiltrating leukocytes (CD5^+^) after different treatments.

**Figure S17.** Typical flow cytometry plots of IFN-γ^+^ in CD8⁺CD3⁺ T cells after different treatments.

**Figure S18**. The ratio of CD8+ T/ Tregs after different treatments.

**Figure S19**. Typical flow cytometry plots of PD-1⁺ cells among CD3⁺ T cells after different treatments.

**Figure S20**. The body weight of B16F10 tumor-bearing mice with different treatments (n = 5).
